# Supplementary material for: The impact of the military conflict in Sudan on maternal health: a mixed qualitative and quantitative study
Source: PeerJ. 2024 Jun 24;12:e17484. doi: 10.7717/peerj.17484 (PMC11210456; doi:10.7717/peerj.17484)
Supplement: Supplemental Information 1 [file peerj-12-17484-s001.docx]

**The impact of the military conflict in Sudan on Maternal health; a mixed qualitative and quantitative study, 2023**

**Qualitative data**

Data was originally in Arabic language

| لقد جعل النزاع من الصعب للغاية الحصول على رعاية ما قبل الولادة والمساعدة الطبية أثناء الولادة. تُجبر العديد من النساء على ولادة أطفالهن في ظروف غير آمنة  كنت حاملاً في الشهر السابع عندما بدأ النزاع. تم إغلاق أقرب مستشفى، ولم أجد وسيلة نقل لأخذي إلى المستشفى التالي. واضطررت إلى الاعتماد على الطب التقليدي، وهو ما لم يكن كافياً. قلقة بشأن صحة طفلي ". |
| --- |
| أشعر بالخوف والقلق الدائمين على سلامة طفلي الذي لم يولد بعد. العنف المستمر يجعل من الصعب العثور على مكان آمن للولادة. ·كان المستشفى مكتظًا، ولم يكن هناك عدد كافٍ من الأسرة. اضطررت إلى مشاركة السرير مع امرأة أخرى ولدت لتوها. كان العاملون في الرعاية الصحية مرهقين ومجهدين للعمل. لم يكن لديهم المعدات اللازمة، ولم تكن الظروف مناسبة. ينظف |
| ادى النزوح وعدم الاستقرار إلى تقييد إمكانية الوصول إلى برامج الرعاية السابقة للولادة، مما حرمني من إجراء فحوصات وفحوصات مهمة. "كنت أخشى مغادرة منزلي بسبب طلقات نارية في الخارج. لم يكن لدي نقود لدفع تكاليف النقل إلى المستشفى. لم أكن أعرف من أطلب المساعدة أو إلى أين أذهب. شعرت بالوحدة الشديدة. |
| إن نقص الإمدادات الطبية وأخصائي الرعاية الصحية المدربين يعرضني أنا وطفلي للخطر. إنه صراع دائم للعثور على الموارد اللازمة لحمل صحي. |
| لقد رأيت العديد من النساء في مجتمعي يعانين من مضاعفات أثناء الولادة بسبب نقص الرعاية الطبية المناسبة. إنه أمر مفجع. |
| لقد أثر الصراع المستمر على صحتي العقلية، وأنا قلق من تأثيره على طفلي. التوتر هائل |
| العيش في مخيمات النزوح المزدحمة دون الحصول على المياه النظيفة والصرف الصحي يعرض النساء الحوامل لخطر أكبر للإصابة بالعدوى والقضايا الصحية الأخرى. ذهبت إلى قابلة تقليدية. لقد ساعدتني في الولادة وتعافيت بشكل جيد. كما تلقيت دعمًا من عائلتي وأصدقائي، مما ساعدني في التغلب عليها." |
| أخشى على سلامتي كلما اضطررت إلى مغادرة منزلي لطلب المساعدة الطبية. مناطق النزاع خطيرة، ومن الصعب البقاء في أمان. كنت حاملاً عندما بدأ النزاع وكنت قلقة على صحة طفلي. تم إغلاق أقرب مستشفى، ولم أجد أي وسيلة نقل لنقلي إلى المستشفى التالي. واضطررت إلى الاعتماد على الطب التقليدي، وهو ما لم يكن كذلك. كافي. كنت خائفة وقلقة على صحة طفلي ". |
| النقل قضية رئيسية. يكاد يكون من المستحيل الوصول إلى مرافق الرعاية الصحية في الوقت المناسب، مما قد يهدد الحياة أثناء حالات الطوارئ. ولدت أثناء النزاع ، وكانت الظروف في المستشفى سيئة للغاية. كان المستشفى مكتظًا ولا توجد أسرة كافية. اضطررت إلى تقاسم السرير مع امرأة أخرى ولدت لتوها. العاملون في مجال الرعاية الصحية مرهقون ومرهقون. لم يكن لديهم المعدات اللازمة والظروف لم تكن نظيفة |
| تفتقر خدمات الدعم والرعاية بعد الولادة في المناطق المتضررة من النزاع، مما يجعلني أشعر بعدم الدعم والضعف بعد الولادة |
| أصبحت تكلفة الرعاية السابقة للولادة والولادة عبئًا. فقد أدى الصراع إلى تفاقم الوضع الاقتصادي، مما جعل من الصعب على تحمل تكاليف الرعاية الصحية اللازمة |
| أواجه التمييز ووصمة العار كامرأة حامل في منطقة نزاع. وهذا يضيف إلى التحديات التي أواجهها بالفعل في الحصول على الرعاية الصحية والدعم |
| المستشفيات المكتظة والموارد المحدودة تجعل من الصعب الحصول على الرعاية المناسبة. نظام الرعاية الصحية غارق في مطالب الصراع |
| أدى الخوف وانعدام الأمن الناجمين عن النزاع إلى زيادة أخطار الولادة المبكرة. ومن دواعي القلق الدائم أن طفلي قد يأتي مبكرًا |
| سمعت عن نساء يفقدن حياتهن أثناء الولادة بسبب عدم توفر قابلات ماهرات. إنها مأساة كان من الممكن منعها |
| أدى الصراع إلى انقطاع إمدادات الأدوية الأساسية، مما جعل من الصعب علي الحصول على العلاجات اللازمة لحمل صحي |
| كثيراً ما تمنعني الأعراف والتقاليد الثقافية من طلب المساعدة الطبية أثناء الولادة، وقد زاد الصراع من صعوبة تحدي هذه الممارسات. |
| نقص المياه النظيفة ومرافق الصرف الصحي يزيد من مخاطر العدوى والمضاعفات. إنه مصدر قلق دائم للنساء الحوامل مثلي |
| ندرة الدم ومشتقاته مصدر قلق دائم. في حالة حدوث مضاعفات، من غير المؤكد ما إذا كانت عمليات نقل الدم اللازمة ستكون متاحة. |
| أصبحت خدمات تنظيم الأسرة غير متاحة، مما أدى إلى حدوث حالات حمل غير مقصود وزيادة الضغط على خدمات صحة الأم |
| الضيق النفسي الناجم عن النزاع يجعل من الصعب تأسيس الرضاعة الطبيعية والحفاظ عليها. إنه صراع بالنسبة لي ولطفلي |
| تسبب النزوح في تحديات إضافية لحملي. من الصعب أن أجد تغذية مناسبة وبيئة مستقرة لضمان صحة طفلي." |
| أدى النزاع إلى تعطيل برامج التثقيف قبل الولادة، مما تركني بمعرفة محدودة حول الرعاية والممارسات المناسبة أثناء الحمل |
| لقد شهدت زيادة في حالات زواج الأطفال وحالات الحمل المبكر في مجتمعي بسبب عدم الاستقرار الناجم عن النزاع. وهو يعرض الأمهات الشابات وأطفالهن لمخاطر أكبر. |
| إن النقص في القابلات الماهرات يجعلني أشعر بالقلق على سلامة ولادتي. أشعر بالقلق من المضاعفات التي قد تنشأ أثناء الولادة |
| يعتبر العنف الجنسي مشكلة منتشرة في مناطق النزاع ، ويمكن أن يكون لعواقبه عواقب وخيمة على النساء الحوامل وأطفالهن |
| أدى تدمير البنية التحتية للرعاية الصحية إلى صعوبة الوصول إلى الرعاية الطبية المناسبة. ومن المحبط أن نرى حالة نظام الرعاية الصحية لدينا |
| الافتقار إلى البنية التحتية الأساسية يزيد من تعقيد الحمل. فبدون الكهرباء والصرف الصحي المناسب ، من الصعب ضمان بيئة صحية لطفلي |
| لقد جعلني تعطيل أنظمة الدعم أشعر بالعزلة والوحدة. من الصعب أن أجد المساعدة والتوجيه الذي أحتاجه خلال هذا الوقت الحرج |
| رعاية التوليد في حالات الطوارئ نادرة في مناطق النزاع ، ومن المخيف التفكير في المضاعفات المحتملة التي قد أواجهها دون مساعدة فورية |
| الحصول على الوثائق الضرورية وتحديد الهوية يمثل تحديًا في منطقة متأثرة بالنزاع ، مما يجعل من الصعب علي الوصول إلى خدمات الرعاية الصحية |
| يضيف النقص في العاملين في مجال الرعاية الصحية إلى الوصول المحدود بالفعل للمساعدة الطبية. إنه لأمر محبط الانتظار لساعات أو السفر لمسافات طويلة للحصول على الرعاية |
| إن وصمة العار والتمييز التي تتعرض لها الناجيات من العنف الجنسي تمنع العديد من النساء من طلب المساعدة التي يحتجن إليها. إنها حقيقة مفجعة |
| أنا قلقه بشأن المستقبل والتأثير طويل المدى للصراع |

"The conflict has made accessing prenatal care and medical assistance during childbirth extremely challenging. Many women are forced to deliver their babies in unsafe conditions. "I was afraid to leave my house because there were gunshots outside. I had no money to pay for transportation to the hospital. I did not know who to ask for help or where to go. I felt very alone."

· "I was six months pregnant when the conflict started. The nearest hospital was closed, and I could not find any transport to take me to the next hospital. I had to rely on traditional medicine, which was not sufficient. I was scared and worried about my baby's health."

· "The hospital was overcrowded, and there were insufficient beds. I had to share a bed with another woman who had just given birth. The healthcare workers were exhausted and overworked. They didn't have the necessary equipment, and the conditions were not clean."

· "I constantly fear and worry about my unborn child's safety. The ongoing violence makes finding a safe place to give birth difficult."

· "The lack of medical supplies and trained healthcare professionals puts me and my baby at risk. It's a constant struggle to find the necessary resources for a healthy pregnancy."

· "I've seen many women in my community suffer from complications during childbirth due to the lack of proper medical care. It's heartbreaking."

· "The ongoing conflict has taken a toll on my mental health, and I worry about the impact it may have on my baby. The stress is overwhelming."

· "Living in crowded displacement camps without access to clean water and sanitation puts pregnant women at a higher risk of infections and other health issues. "I was afraid to leave my house because there were gunshots outside. I had no money to pay for transportation to the hospital. I did not know who to ask for help or where to go. I felt very alone. I went to a traditional birth attendant. She helped me with delivery and recovered well. I also had the support of my family and friends, which helped me get through it.

· "I fear for my safety whenever I leave my home to seek medical assistance. The conflict zones are dangerous, and staying safe is a constant struggle.

I was pregnant when the conflict started and worried about my baby's health. The nearest hospital was closed, and I could not find any transport to take me to the next hospital. I had to rely on traditional medicine, which was not sufficient. I was scared and worried about my baby's health. *I was afraid of leaving my house because there were gunshots outside.*

· "I've heard of women losing their lives during childbirth due to the unavailability of skilled birth attendants. It's a tragedy that could have been prevented."

· "The conflict has disrupted the supply of essential medications, making it difficult for me to receive the necessary treatments for a healthy pregnancy."

· "Cultural norms and traditions often prevent me from seeking medical help during childbirth, and the conflict has made it even harder to challenge these practices."

· "The lack of clean water and sanitation facilities increases the risk of infections and complications. It's a constant worry for pregnant women like me."

· "Displacement has brought additional challenges to my pregnancy. It's hard to find proper nutrition and a stable environment to ensure my baby's health."

· "Transportation is a major issue. It's almost impossible to reach healthcare facilities in a timely manner, which could be life-threatening during emergencies."

· "The conflict has disrupted prenatal education programs, leaving me with limited knowledge about proper care and practices during pregnancy."

· "I've witnessed an increase in child marriages and early pregnancies in my community due to the instability caused by the conflict. It puts young mothers and their babies at higher risks. I gave birth during the conflict, and the conditions at the hospital were very poor. The hospital was overcrowded, and there were insufficient beds. I had to share a bed with another woman who had just given birth. The healthcare workers were exhausted and overworked. They didn't have the necessary equipment, and the conditions were not clean.

· "The shortage of skilled birth attendants makes me anxious about the safety of my delivery. I worry about complications that may arise during childbirth."

· "Sexual violence is a prevalent issue in conflict zones, and the aftermath can have severe consequences for pregnant women and their babies."

· "The destruction of healthcare infrastructure has made it difficult to access proper medical care. It's disheartening to see the state of our healthcare system."

· "Displacement and instability have limited my access to antenatal care programs, depriving me of important screenings and check-ups."

· "The scarcity of blood and blood products is a constant concern. In case of complications, it's uncertain whether the necessary transfusions will be available."

· "Family planning services have become inaccessible, leading to unintended pregnancies and added strain on maternal health services."

· "The psychological distress of the conflict makes establishing and maintaining breastfeeding challenging. It's a struggle for both me and my baby."

· "Postnatal care and support services are lacking in conflict-affected areas, leaving me feeling unsupported and vulnerable after giving birth."

· "The cost of prenatal care and childbirth has become a burden. The conflict has worsened the economic situation, making it harder for me to afford the necessary healthcare."

· "I face discrimination and stigma as a pregnant woman in a conflict zone. It adds to my challenges in accessing healthcare and support."

· "Overcrowded hospitals and limited resources make it difficult to receive adequate care. The healthcare system is overwhelmed by the demands of the conflict. *I gave birth during the conflict, and the conditions at the hospital were very poor. The hospital was overcrowded, and there were insufficient beds. I had to share a bed with another woman who had just given birth. The healthcare workers were exhausted and overworked. They didn't have the necessary equipment, and the conditions were not clean.*

· "The fear and insecurity caused by the conflict have increased my risk of preterm birth. It's a constant worry that my baby might come too soon."

· "The lack of basic infrastructure further complicates my pregnancy. Without electricity and proper sanitation, it's hard to ensure a healthy environment for my baby."

· "The disruption of support systems has left me isolated and alone. It's hard to find the help and guidance I need during this critical time."

· "Emergency obstetric care is scarce in conflict zones, and it's terrifying to think about the potential complications I might face without immediate assistance."

· "Obtaining necessary documentation and identification is challenging in a conflict-affected area, making it harder for me to access healthcare services."

· "The shortage of healthcare workers adds to the already limited access to medical assistance. It's frustrating to wait hours or travel long distances for care."

· "The stigma and discrimination faced by survivors of sexual violence prevent many women from seeking the help they need. It's a heartbreaking reality."

· "I worry about the future and the long-term impact of the conflict on my child's health. It's a constant burden on my mind."
