# Supplementary material for: The impact of the military conflict in Sudan on maternal health: a mixed qualitative and quantitative study
Source: PeerJ. 2024 Jun 24;12:e17484. doi: 10.7717/peerj.17484 (PMC11210456; doi:10.7717/peerj.17484)
Supplement: Supplemental Information 3 [file peerj-12-17484-s003.docx]

**تأثير الصراع العسكري في السودان على صحة الأم. دراسة نوعية وكمية مختلطة، 2023**

أنتم مدعوون للمشاركة في دراسة بحثية حول تأثير الصراع العسكري في السودان على صحة الأم. تهدف هذه الدراسة إلى فهم آثار الصراع العسكري على صحة الأمومة للمرأة في السودان. إن مشاركتك في هذه الدراسة تطوعية تمامًا، ونود أن نزودك بمعلومات حول الدراسة قبل أن تقرر ما إذا كنت ستشارك أم لا.

**د. الهادي مسكين**

**أستاذ مساعد – قسم امراض النساء والتوليد- جامعة بيشة / جامعة الجزيرة**

**هل أنت موافق؟**

نعم. لا

**موافقتك مطلوبة للانتقال إلى الخطوة التالية.**

1. الرقم.........................................
2. العمر ........................................... ......................... (سنين)
3. عدد الأطفال
4. سهولة الوصول إلى الرعاية الصحية.

أ. نعم.

ب. لا

1. طريقة الولادة

أ. الولادة المهبلية.

ب. العملية القيصرية

1. هل عانيتي من أي مضاعفات ولادية

أ. نعم

ب. لا

1. التعليم

أ. لا يوجد تعليم رسمي

ب. مدرسة ابتدائية

ج. المدرسة الثانوية

د. كلية جامعية او دراسات عليا

1. عمر الحمل ........................................... ........أسابيع
2. امكانية سهولة الوصول إلى الرعاية الصحية

أ. نعم.

ب. لا

**السؤال المفتوح المستخدم للبيانات النوعية:**

من فضلك، هل يمكنك إخبارنا عن تجربتك مع إمكانية الوصول والتحديات المتعلقة بالجودة في خدمات صحة الأم؟
